# Vibrotactile Presentation of Musical Notes to the Glabrous Skin for Adults with Normal Hearing or a Hearing Impairment: Thresholds, Dynamic Range and High-Frequency Perception

**DOI:** 10.1371/journal.pone.0155807

**Published:** 2016-05-18

**Authors:** Carl Hopkins, Saúl Maté-Cid, Robert Fulford, Gary Seiffert, Jane Ginsborg

**Affiliations:** 1 Acoustics Research Unit, School of Architecture, University of Liverpool, Liverpool, United Kingdom; 2 Centre for Music Performance Research, Royal Northern College of Music, Manchester, United Kingdom; University of Chicago, UNITED STATES

## Abstract

Presentation of music as vibration to the skin has the potential to facilitate interaction between musicians with hearing impairments and other musicians during group performance. Vibrotactile thresholds have been determined to assess the potential for vibrotactile presentation of music to the glabrous skin of the fingertip, forefoot and heel. No significant differences were found between the thresholds for sinusoids representing notes between C1 and C6 when presented to the fingertip of participants with normal hearing and with a severe or profound hearing loss. For participants with normal hearing, thresholds for notes between C1 and C6 showed the characteristic U-shape curve for the fingertip, but not for the forefoot and heel. Compared to the fingertip, the forefoot had lower thresholds between C1 and C3, and the heel had lower thresholds between C1 and G2; this is attributed to spatial summation from the Pacinian receptors over the larger contactor area used for the forefoot and heel. Participants with normal hearing assessed the perception of high-frequency vibration using 1s sinusoids presented to the fingertip and were found to be more aware of transient vibration at the beginning and/or end of notes between G4 and C6 when stimuli were presented 10dB above threshold, rather than at threshold. An average of 94% of these participants reported feeling continuous vibration between G4 and G5 with stimuli presented 10dB above threshold. Based on the experimental findings and consideration of health effects relating to vibration exposure, a suitable range of notes for vibrotactile presentation of music is identified as being from C1 to G5. This is more limited than for human hearing but the fundamental frequencies of the human voice, and the notes played by many instruments, lie within it. However, the dynamic range might require compression to avoid the negative effects of amplitude on pitch perception.

## Introduction

This paper reports two experiments, the findings of which demonstrate the potential for vibrotactile presentation of music to the glabrous skin of the fingertip, forefoot and heel. This informs the development of vibrotactile technology to be used by musicians with hearing impairments to facilitate interaction with other musicians when improvising, rehearsing and performing together, as well as to open up new opportunities for people with hearing impairments to make music with other musicians. The research was inspired by Dame Evelyn Glennie, the highly-renowned solo percussionist who is classified as profoundly deaf with residual hearing at very high amplification [1]. Many of the percussion instruments that she plays cause the floor to vibrate and this vibration can be transmitted from the floor through her bare feet into her body. Fulford *et al* [2] showed that the main challenges for amateur and professional musicians with hearing impairments are staying in time and staying in tune when performing with other musicians. Group improvisation, rehearsal and performance for musicians with hearing impairments could be facilitated by technology to transmit different vibration signals simultaneously to different musicians. Vibration can be transmitted to the fingertips for singers, while players of instruments would require some form of vibrating performance deck for the feet or vibration pads attached to the body. Vibrotactile stimuli could also augment auditory information for musicians who use hearing aid technology or cochlear implants; whilst these devices increase the audibility of music they can also distort it because they are primarily designed for speech (e.g. see [3,4]). Vibrotactile feedback would therefore augment any auditory information. Finally, musicians with normal hearing who use amplification when performing could use vibrotactile feedback to supplement the use of in-ear or floor monitors at lower listening levels, thus reducing the risk of noise induced hearing loss.

The first experiments to determine the extent to which “hearing through the skin” is possible were reported by Gault [5] in 1926. Since then, research has primarily focussed on the potential for deaf people to monitor their own voices and understand other people’s speech. While Gault expressed interest in vibrotactile perception for the appreciation of music in at least “the lower and middle reaches of the musical scale” he carried out no experimental work on this topic. Gault and Crane [6] explored the vibrotactile representation of speech using a device called a ‘teletactor’, which had a vibrator for each finger of the hand. Knudsen [7] approached the physics of vibrotaction by creating an apparatus to apply vibration stimuli to the finger but was pessimistic about the potential for music to be presented in this way stating that it “would be almost void of melody or pitch coloring”. In 1960, Geldard [8] discussed what was by then current thinking on the use of the skin as a communication channel. He argued that associations between vibratory frequency and pitch are tenuous and uncertain because vibrotactile pitch perception depends on amplitude as well as frequency, and concluded that the skin cannot be used to discriminate speech frequencies. However, recent experiments using voice coils embedded in the back of a chair do indicate the potential to present speech and musical information as vibrotactile stimuli: Ammirante *et al* [9] found that participants with hearing impairments are able to discriminate between same-sex talkers and Russo *et al* [10] demonstrated that participants with and without hearing impairments can discriminate musical timbre. Whilst voice coils are not ideal for precise, controlled presentation of vibrotactile stimuli (partly because of the unknown damping by the skin or clothing [11]), the findings indicate that the human integument is able to distinguish subtleties of timbre. Hence it is worthwhile assessing whether vibrotactile stimuli can provide information that is sufficient for the requirements of musicians playing together.

As a first stage in developing an effective method for presenting music in the form of vibrotactile stimuli, the present study aimed to determine vibrotactile detection thresholds for the glabrous skin at three locations on the body chosen to cause as little interference to the demands of singing and/or playing a musical instrument: (1) the fingertip, i.e. on the surface of the whorl, arch or loop on the distal phalanx of the middle finger, (2) the forefoot, i.e. on the distal part of the plantar side of the foot involving the distal and proximal phalanxes and partially the metatarsals and (3) the heel, i.e. on the proximal part of the plantar side of the foot, underneath the calcaneous bone. Singers can use both hands and feet for vibrotactile feedback, but players of instruments are more likely to have to use their feet. However the potential for using the heel and forefoot of both feet opens up the possibility for all musicians to monitor not only their own output, but also that of others’ voices or instruments played by co-performers. The choice of glabrous skin of the hands and feet, rather than hairy skin (e.g. volar forearm) is that glabrous skin is more sensitive than hairy skin, so detection thresholds are likely to be lower [12,13]. The perception of vibration on the glabrous skin is mediated by a Pacinian channel and three non-Pacinian channels for which suprathreshold stimulation can simultaneously activate two or more of these channels [14]. Pacinian mechanoreceptors are found deep beneath the glabrous skin of the hands and feet, and are distributed fairly evenly over the glabrous area [15]. They are capable of temporal and spatial summation and typically respond to vibration stimuli between 40 and 800Hz [14]. Vibrotactile thresholds are usually determined for the purposes of investigating the responses of mechanoreceptors (e.g. [16,17]), particularly on specific, small areas of the body for diagnostic assessments of nerve dysfunction [18]. A small contactor area and contactor surround are used to minimise the propagation of surface waves beyond the contactor surround that may stimulate mechanoreceptors on areas of the skin further away from the excitation point. However, with or without a contactor surround, vibrotactile thresholds are determined by both Pacinian and non-Pacinian channels [19]. When there is no contactor surround the area stimulated is larger so the thresholds for the Pacinian channel, which is capable of spatial summation, can be lowered, and the thresholds for the non-Pacinian channels can be raised [19]. As the present study investigated the potential for vibrotactile feedback with group performance, relatively large contactors without surrounds were used to determine vibrotactile thresholds. Establishing thresholds using small contactors with surrounds would not be relevant to singers or players (a) receiving vibrotactile information derived from the output of their own voice or instrument, or other musician’s instruments (particularly for signals received from a large surface or object such as a vibrating platform designed to enable the musician to easily make or interrupt contact with it during performance) or (b) feeling vibration on the surface of their own musical instrument.

Vibrotactile thresholds are usually determined between 4Hz and 160Hz for diagnostic purposes [18], although psychophysical studies on mechanoreceptors tend to measure only up to 500Hz (e.g. [17]) or 700Hz (e.g. [20]). Geldard noted in his review of vibrotactile frequency responses [21] that the highest perceptible frequency of vibration was not known with certainty and could potentially lie between 640Hz and 2600Hz. The highest frequency measured in experiments on Pacinian corpuscles taken from cat mesentery or monkey nerve fibres is 1.5kHz [22]. Since the present study investigated the potential for vibrotactile presentation of music, the pitch ranges of the human voice and the most commonly used musical instruments informed the decision as to which frequencies would be used in the experiments. The range of the typical 88-key piano is from A0 (27.5Hz) to C8 (4186Hz), but the fundamental frequencies of the human voice and instruments including lower bowed strings (viola, cello, double bass), plucked strings (banjo, six-stringed and bass guitar), lower woodwind (cor anglais, saxophone, bassoon), brass (trumpet, French horn, trombone, tuba) all lie within the range from C1 (32.7Hz) to C6 (1046.5Hz). This range was therefore chosen for the experiments.

The aims of the study were to (1) establish and compare vibrotactile thresholds on the fingertips, forefeet and heels of participants with normal hearing, using contactor of sizes appropriate to music performance; (2) determine potential differences between vibrotactile thresholds on the fingertips of participants with and without hearing impairments (categorised as severe or profound hearing loss); (3) investigate vibrotactile perception of the continuous and transient parts of high-frequency notes (G4 to C6) on the fingertip; and (4) use the findings of the two experiments that were conducted to assess the potential for the vibrotactile presentation of music in terms of the range of musical notes that can be perceived and the available dynamic range. Each stimulus lasted 1s, corresponding to a minim (half note) at 120bpm. Experiment I determined vibrotactile thresholds and Experiment II assessed the vibrotactile perception of high-frequency notes.

## Methods

### Experiment I. Vibrotactile thresholds

In medical contexts, vibrotactile thresholds are typically determined using a psychophysical testing procedure involving an up-down algorithm, the Békésy algorithm or a forced-choice method. Gandhi *et al* [23] note that there is no consensus on the recommendation of one particular procedure. However, ISO 13091–1 [18] permits the use of both up-down and Békésy algorithms but states that the former is preferred. In the present work, the procedure to determine vibrotactile thresholds was adapted from audiometric procedures to determine thresholds for air conduction, and was based on the shortened version of the ascending method in ISO 8253–1 [24] using an up-down algorithm. Its main advantage is that it is well-established, and therefore likely to be familiar to members of the general public taking part in the study, especially those with hearing impairments. The audiometric procedure uses discrete steps in level of 5dB HL which would give insufficient resolution for vibrotactile thresholds; hence steps of 2dBV were used. The procedure was programmed in Matlab to automate the presentation of stimuli to the participant and the data collection.

Verrillo [25] has shown that, according to Zwislocki’s theory of temporal summation [26], there is no change in vibrotactile thresholds for bursts of sinusoids lasting 1s or more, at and below 500Hz, although thresholds are higher when they are shorter than 1s. In the present study each signal was a sinusoid of 1s duration corresponding to a minim (half note) at 120bpm. In practice, the actual duration was varied between 0.994s and 1.009s depending on the frequency of the note to ensure that there were no abrupt truncations at the beginning and/or end of the sinusoid.

Each stimulus comprised a sequence of signals as follows: the 1s signal presented three times in a row, with each note separated by a 2s interval without any signal. Participants were instructed to press a response button whenever they felt a note. Eleven notes were presented as stimuli corresponding to the musical notes C and G in the range from C1 to C6. The frequencies were calculated using the ratio 2^1/12^ in twelve-tone equal temperament to give the frequency of the *n*th piano key relative to A4. For example, C1 corresponded to 32.7Hz, C4 to 261.6Hz and C6 to 1046.5Hz. The starting tone in audiometric tests is 1kHz, which is the frequency range of highest sensitivity for the human ear. In the present study C4 was chosen as the starting note because it approximately corresponds to the frequency at which the Pacinian corpuscle is most sensitive [16]. The order of presentation began with C4 ascending up to C6, followed by notes descending from G3 to C1.

Verrillo and Bolanowski [27] showed that vibrotactile thresholds for the thenar eminence and the volar forearm vary with temperature above 100Hz, typically with a decrease in the threshold with increasing temperature. Participants’ skin temperatures were monitored at ≈20 minute intervals using an infra-red thermometer (Tenma Type 72–6700). Based on Verrillo and Bolanowski’s findings, the temperature ranges for valid measurements were deemed acceptable between 20 to 36°C for notes C1 to G2 and between 24 to 36°C for notes C3 to C6. If participants’ measured temperature was outside these ranges then the experiment was interrupted until it returned within range.

The familiarisation stage of the experiment was as follows: (a) the stimulus C4 was presented at a maximum level that could be felt by all participants; (b) the level of the stimulus was then reduced in steps of 20dBV until no response was elicited; (c) the level of the stimulus was then increased in 2dBV steps until a response was elicited; (d) the stimulus was then presented again at the maximum level. This was followed by Stage 1 in which the stimulus was presented 10dBV below the lowest level at which it had elicited a response in the familiarisation stage. The stimulus was presented again at levels increasing by steps of 2dBV until a response was elicited. In Stage 2, the stimulus was presented 10dBV below the lowest level at which it had elicited a response in Stage 1 and another ascent was started. This process continued for a maximum of three ascents (excluding the familiarisation stage) until two responses were elicited by the stimulus presented at the same level. The next stimulus was then presented using the same procedure starting from the familiarisation stage and ending with Stage 2 when two responses were elicited by the stimulus presented at the same level. Once all 11 notes had been presented, a verification stage was carried out in which the note C4 was used as the stimulus and presented stepwise as described above. If the level at which C4 elicited a response on this second occasion was within ±4dBV of the first measurement and the participants’ skin temperature had remained within the acceptable range during the whole task, the measurement was deemed valid and the results were included in the analysis. Otherwise, the result was deemed to be invalid and the results were not processed.

A complete test for each participant lasted ≈1.5 hours including ≈15 minutes to brief the participant on the procedure and two or three rest periods lasting ≈5 minutes following each ≈20 minute period of testing.

### Experiment II. Perception of high-frequency vibration using the fingertip

This experiment investigated the perception of continuous and transient parts of high-frequency notes using fingertips. The impetus was that, in pilot testing, the researchers found it difficult to perceive continuous vibration consistently for C6 but they could feel a transient at the beginning and/or end of the sinusoidal signal. Using the same procedure as Experiment I, thresholds were determined on the fingertip for the 11 white notes between G4 (392Hz) and C6 (1046.5Hz). The order of presentation began with F5 (698.5Hz) ascending to C6, followed by notes descending from E5 to G4. The note F5 was used in the verification stage rather than C4. When the threshold for a note had been established, the participant was presented with the sequence of signals at threshold level and two-alternative forced choice questions were used to ask if they felt (a) transient vibration at the beginning and/or end of any of the 1s notes in the sequence and (b) continuous vibration during any of the 1s notes in the sequence. The same sequence of signals was then presented 10dB above threshold, because vibration would usually be presented above threshold for vibrotactile presentation of music, and the questions were repeated before proceeding to the next note.

### Test environment and equipment

**Fig 1:**
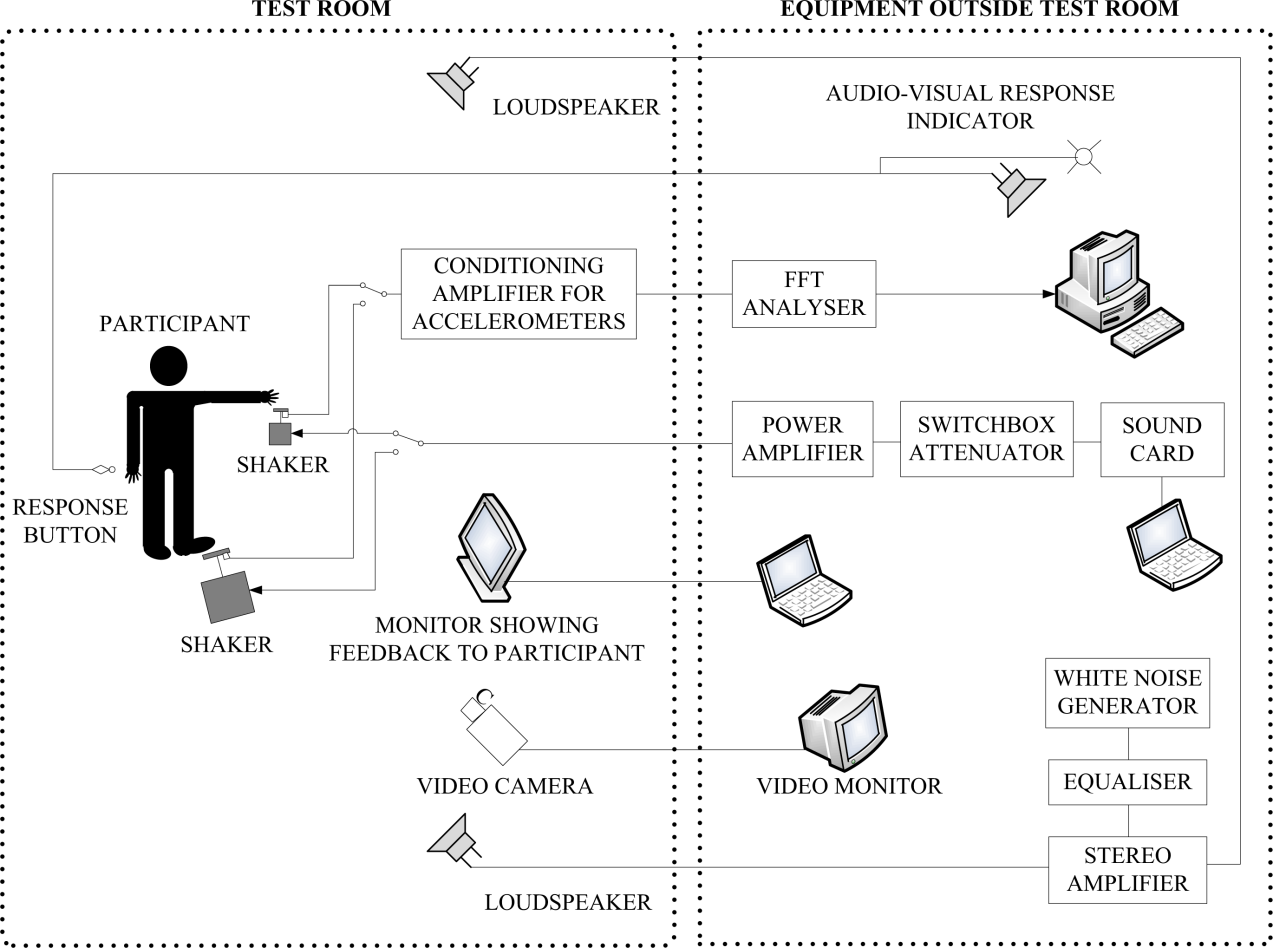
Experimental set-up for Experiments I and II.

All tests on the fingertips and feet were carried out inside an audiometric booth and a semi-anechoic chamber respectively. These environments provided vibration isolation from the rest of the building, had low background noise and avoided visual distractions for the participants as there were no windows. The experimental set-up is shown in Fig 1.

#### Tests on the fingertip

**Fig 2:**
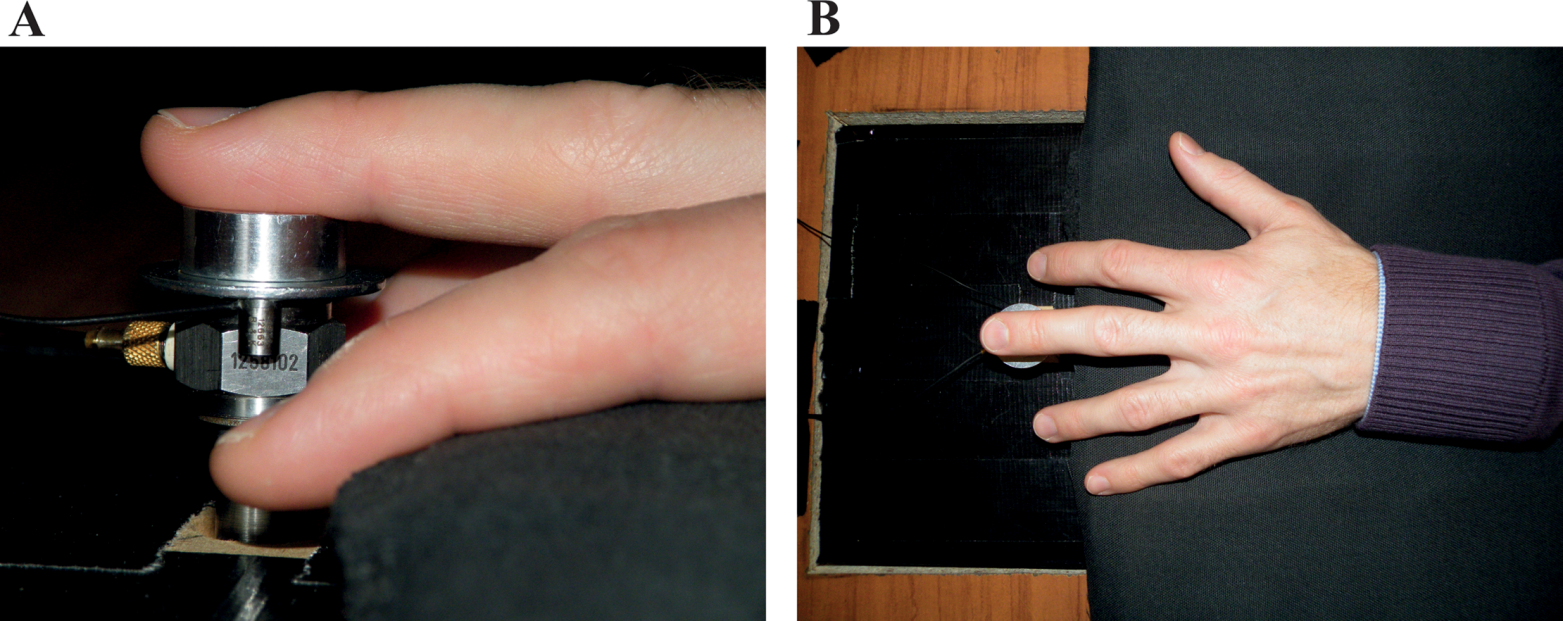
Distal phalanx of the middle finger (A) placed on the contactor and (B) showing hand position.

The contactor used for the fingertip was a circular aluminium disc with a diameter of 2cm (area: 3.14cm^2^). The distal phalanx of the middle finger of the participant’s dominant hand rested upon the contactor as indicated in Fig 2 with the fingertip placed on the contactor such that the whorl, arch or loop of the fingerprint was positioned at the centre of the disc. Participants were instructed to relax their arm and hand on the foam-covered table and not press down upon the contactor.

The contactor height was such that the middle finger rested upon it naturally. The surface roughness of the contactor disc was estimated to have a centre line average of 3.175μm using the procedure in ISO 4288 [28].

The contactor was driven by an electro-dynamic shaker (LDS Type V200) that was structurally isolated from the table upon which the participants arm was supported. This shaker was enclosed in a box to reduce the radiated sound. The displacement of the contactor was measured using an accelerometer (B&K Type 4374) and FFT analyser (Siglab Type 20/42). The static load of the finger on the contactor did not alter its response by more than 0.5dB for notes C3 to C6. The power amplifier introduced very low-level harmonic distortion such that harmonic peaks were at least 40dB below the fundamental frequency used as the test note. System linearity was checked by ensuring that there was no distortion of the waveform in the time domain.

Steps were taken to ensure that the participant perceived vibration only from the contactor. Firstly, the shaker was isolated from the floor of the booth by supporting it on heavy concrete blocks on top of resilient material. Secondly, the shaker was isolated from the table by a hole in the centre of the table so that there was no physical connection between the shaker and the table top. Thirdly, the elbow and forearm of the participant was supported by soft foam on top of the table.

#### Tests on the forefoot and heel

The contactors used for the forefoot and the heel were 2.5cm thick Perspex discs with a diameter of 12cm and 10cm respectively. The middle of the heel was positioned at the centre of the contactor, and the forefoot was positioned so that all the toes were on the contactor. The surface roughness of the contactor discs was estimated to have a centre line average of 1.6μm using the procedure in ISO 4288 [28]. Participants removed their shoe and sock, and rolled long trousers up to the knee, or hitched any long skirt/dress up to the knee, to avoid any sensation from clothes moving near the foot.

The displacement of each contactor was determined from the acceleration measured using an accelerometer (B&K Type 4393) and FFT analyser (Siglab Type 20/42). The contactors for the feet were much larger than for the fingertip which led to non-uniform displacement over the surface of the contactor. However, this variation was negligible below C5 and had a maximum variation of ≈2dB over the contactor surface for notes at and between C5 and C6. The power amplifier introduced very low-level harmonic distortion such that harmonic peaks were at least 40dB below the fundamental frequency and there was no distortion of the waveform in the time domain.

**Fig 3:**
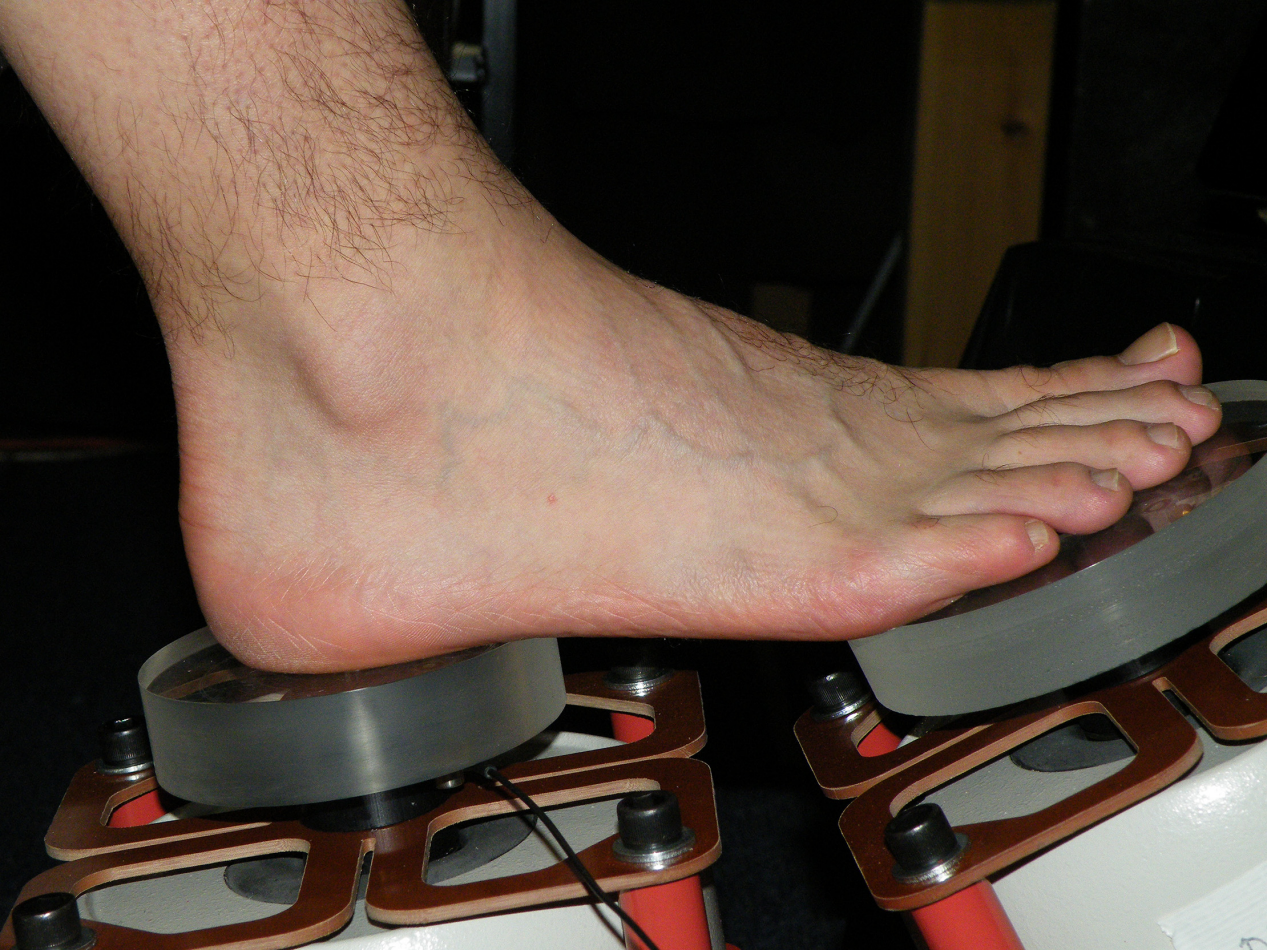
Electrodynamic shakers and contactor discs used to present vibration to the forefoot or the heel.

Each contactor was driven by an electro-dynamic shaker (LDS Type V406 M4-CE) with an auxiliary suspension system to bear the static load of the participant’s leg. For ergonomic purposes, the shakers were inclined at approximately 10° and 25° to the horizontal for the heel and forefoot respectively as indicated in Fig 3. Each shaker was mounted on resilient material to ensure that there was no significant transmission of vibration between the two shakers. Participants were instructed to relax and not press down on the contactor. However it was still necessary to consider the effect of the static load of the leg on the contactor response. This effect was only significant from C1 to C2 for the forefoot and from C1 to C3 for the heel. Therefore a correction was determined for each participant to adjust the signal amplification before the experiment to ensure that the contactor disc response was the same for all participants.

The participant’s seat was isolated from the floor upon which the shakers were supported by using resilient material under the legs of the pedestal that supported the participant. This ensured that any vibration from the shaker was at least 60dB below the levels presented directly to the heel or forefoot.

#### Masking noise

Verrillo and Capraro [29] showed that audible airborne sound from a shaker can significantly affect the threshold. For this reason, hearing defenders and/or auditory masking are needed. Masking noise was used in the present study to avoid any potentially confounding effects from air conduction due to sound radiated by the shaker and the contactor.

While participants undertook the tests on the fingertips, forefoot and heel, broadband noise was presented via two loudspeakers that were positioned symmetrically in front of, and to the left and right sides of the participant; these were orientated at an angle pointing towards the participant’s ears. For the forefoot and heel, the shaker and contactors were significantly larger than for the fingertip and therefore were more efficient at radiating sound. For this reason a graphic equaliser was used to increase the masking noise levels below 100Hz. However, in both cases the sound pressure level measured at the participant’s head was ≈68dB *L*_Aeq_.

### Participants

Approval for the research was granted by the Research Ethics Sub-Committee for Non-Invasive Procedures at the University of Liverpool. Participants who did not have any self-reported impairment of sensation in their hands or feet (i.e. no indication of neuropathy) and who agreed to take part in the study gave their informed consent by signing a consent form approved by the committee.

Two groups of participants were recruited, one group with normal hearing and the other with hearing impairments self-reported according to one of the classifications provided by the charity Action on Hearing Loss [30] (formerly known as the Royal National Institute for the Deaf): mild, moderate, severe or profound hearing loss. People with mild hearing loss can have some difficulty following speech, mainly in noisy situations. The quietest sounds they can hear average between 25dB and 39dB. With a moderate hearing loss, people may have difficulty following speech without hearing aids and the quietest sounds they can hear average between 40dB and 69dB. People with severe hearing loss rely to a great extent on lip-reading, even with hearing aids, and the quietest sounds they can hear average between 70dB and 94dB. British Sign Language (BSL) may be their first or preferred language. For people who are profoundly deaf, BSL may be the first or preferred language, or they might communicate by lip-reading. The quietest sounds they can hear average 95dB or more.

#### Experiment I (fingertip)

Forty-one participants with self-reported normal hearing were recruited, but valid results were obtained from only 32 participants (13 female and 19 male) whose ages ranged from 18 to 65 years (mean: 30.6, SD: 9.2); 21 participants were aged between 18 and 30, ten participants were aged between 31 and 50 and one participant was aged 65 years. All but one of the participants were right-handed, and all carried out the experimental tasks using the fingertip of the middle finger of the right hand.

Eleven participants with severe or profound hearing loss were recruited (only a few people with mild or moderate hearing loss were available), but valid results were obtained from only eight participants (six female and two male) whose ages ranged from 23 to 67 years (mean: 44, SD: 15.8); two participants were aged between 23 and 30, three participants were aged between 31 and 50 years and three participants were aged between 51 and 67 years. All were right-handed and carried out the experimental tasks using the fingertip of the middle finger of the right hand.

#### Experiment I (forefoot and heel)

Twenty-nine participants with self-reported normal hearing were recruited. Twenty (ten female and ten male) took part in tests using the heel of the right foot from which valid results were obtained from all participants. Twenty (ten female and ten male) took part in tests using the forefoot of the right foot from which valid results were obtained from 17 participants. Their ages ranged from 17 to 57 years (mean: 31.7, SD: 11.4); 17 participants were aged between 17 and 30 years, ten participants were aged between 31 and 50 years and two participants were aged between 51 and 57 years. Their foot size (UK system) was in the range 4 to 15.5 (mean: 7.2, SD: 2.6); their weight was in the range 42 to 125kg (mean: 66.3, SD: 16.5); and their height was in the range 1.5 to 2.0m (mean: 1.7, SD: 0.1).

#### Experiment II (fingertip)

Fourteen participants with self-reported normal hearing were recruited from those who took part in Experiment I (fingertips). They were aged 25 to 65 years (mean: 34.7, SD: 10.8); seven participants were aged between 25 and 29 years, three were aged between 33 and 34 years, two were aged between 40 and 41 years, one was aged 50 years and one was aged 65 years. They were all right-handed and carried out the experimental tasks using the fingertip of the middle finger of the right hand.

### Statistical methods

Statistical tests were used to examine the significance of group differences. The normality of the data was assessed using the Shapiro-Wilk test. Comparisons of datasets were then made using either parametric (independent *t*-test) or non-parametric analysis (Mann-Whitney test) for distributions that were assessed to be normal or non-normal respectively. Effect size was assessed using the *r*-value which represents a standardized effect size that can be used to identify a small, medium or large effect.

## Results

### Experiment I (fingertip)

**Fig 4:**
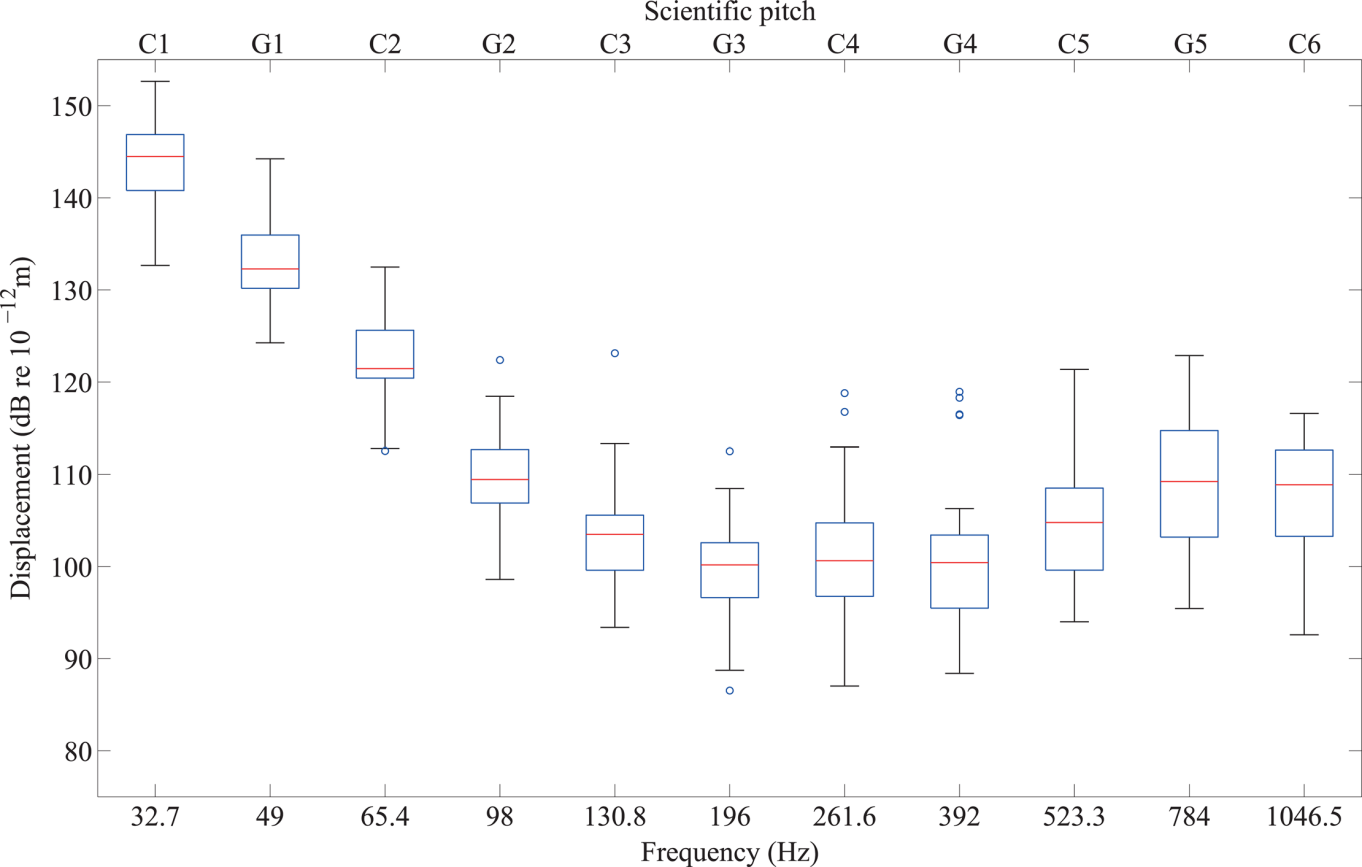
Vibrotactile thresholds on the fingertip from 32 participants with normal hearing for notes between C1 and C6. The median is indicated by the red line. The 25th and 75th percentiles of the values form the lower and upper bounds of the blue box which contains the middle 50% of the values. The black whiskers indicate values within ±3 standard deviations from the mean and the blue circles represent the outliers outside this range.

The vibrotactile thresholds of 32 participants with normal hearing for 11 notes across five octaves (C1 to C6) are shown in Fig 4. For notes from C1 to C6 presented to the fingertip, the curves generally follow the characteristic threshold curve for responses mediated by the Pacinian corpuscle [31] when plotting displacement in decibels using a logarithmic frequency scale. When considering displacement, the lowest threshold typically occurs between 200Hz and 300Hz (e.g. see [16,17]). According to the measurements made in the present study, the lowest thresholds were evident between G3 (196Hz) and C4 (262Hz). Six participants (three male, three female, age range: 21 to 38 years) had responses for one, two or three notes out of the eleven notes that were identified as outliers. This resulted in a total of 12 outliers. However, there was no reason to justify excluding a few data points from these particular participants’ dataset when their results for other notes were not outliers. It was found that C6 was the only note that could not be felt by some participants; of the 32 participants with normal hearing, five could not feel C6, and of the eight participants with a severe or profound hearing loss, there was one participant who could not feel C6.

**Fig 5:**
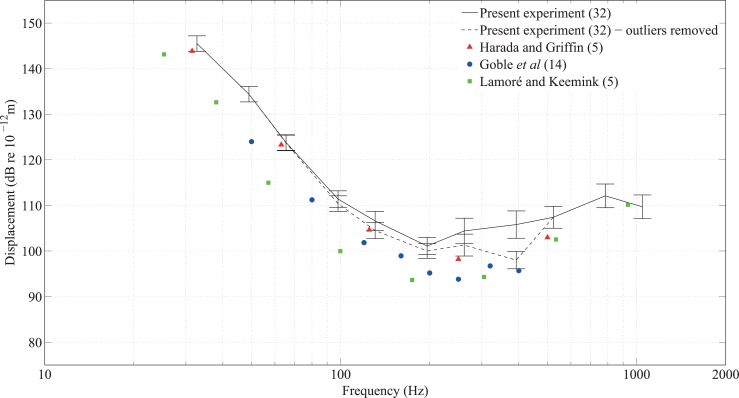
Comparison of vibrotactile thresholds on the fingertip from the present experiment with other published data without a contactor surround from participants with normal hearing. The number of participants is indicated in brackets. The results from the present experiment are shown as mean values with 95% confidence intervals.

To assess the validity of the dataset, the mean thresholds of these participants (including and excluding outliers) were compared with thresholds on the fingertip reported in the literature by Lamoré and Keemink [32], Goble *et al* [11], and Harada and Griffin [33], who–like the present investigators–tested without a contactor surround. These results are shown in Fig 5. Similar thresholds were obtained for the same frequencies, although those found by Goble *et al* and Lamoré and Keemink are lower than in the present study. The differences found between the thresholds for the fingertip in the present study and those found by other investigators can partly be attributed to the size of contactor area and, in some cases, the duration of stimuli as well as the number and age of participants. Harada and Griffin’s contactor area was 0.39cm^2^, Goble *et al*’s was 1.4cm^2^ and Lamoré and Keemink’s was 1.5cm^2^, all notably smaller than the 3.14cm^2^ contactor area used in the present experiment. The duration of the signals in the present experiment (1s) was the same as in Lamoré and Keemink’s study but longer than in Goble *et al* (0.5s). Harada and Griffin tested five participants (age range 23–28 years), Goble *et al* tested 14 participants (age range 18–30 years) and Lamoré and Keemink tested five participants (age range not stated). Fig 5 shows similarly shaped threshold curves but the results from Goble *et al* and Lamoré and Keemink tend to be lower than in the present experiment. This could be explained by the choice of psychophysical procedure because the present experiment uses an up-down algorithm whereas Goble *et al* and Lamoré and Keemink used a forced choice method. The latter approach has previously been found to result in thresholds that are up to 6dB lower [34]. (Note that the psychophysical procedure used by Harada and Griffin is not described in their paper.)

The findings of the present experiment, regardless of outliers, agree most closely with those of Harada and Griffin even though they used a contactor with a much smaller area. The threshold is inversely proportional to the contactor area with [16] or without [19] a contactor surround, although the effect is significantly reduced for the latter [19]. Measurements on the thenar eminence without a contactor surround by Gescheider *et al* [19] showed a 6dB decrease in the threshold when changing from a 0.2cm^2^ to a 3cm^2^ contactor area, a finding that was attributed to spatial summation by the Pacinian receptors. Laser vibrometry confirms that in the absence of a contactor surround, surface waves can propagate from the fingertip along the finger without significant decay [35]; hence these waves could elicit responses from Pacinian receptors further from the excitation areas on the fingertip. Despite the difference in contactor area, the 95% confidence intervals from the present experiment (with or without outliers) tend to encompass the mean values from Harada and Griffin. Comparisons by Morioka and Griffin [36] for the fingertip with a contactor surround with previous studies have also noted that whilst the threshold curves have similar shapes, they vary in their absolute values due to different experimental and psychophysical procedures. More participants took part in Experiment I than in previous studies so it is difficult to draw conclusions from comparisons of thresholds obtained using different sizes of contactor. Whilst the comparison between the findings of the present experiment with previous studies suggested no reason to exclude outliers when calculating mean thresholds, the median was nonetheless selected as a more robust measure in the remaining analyses.

**Fig 6:**
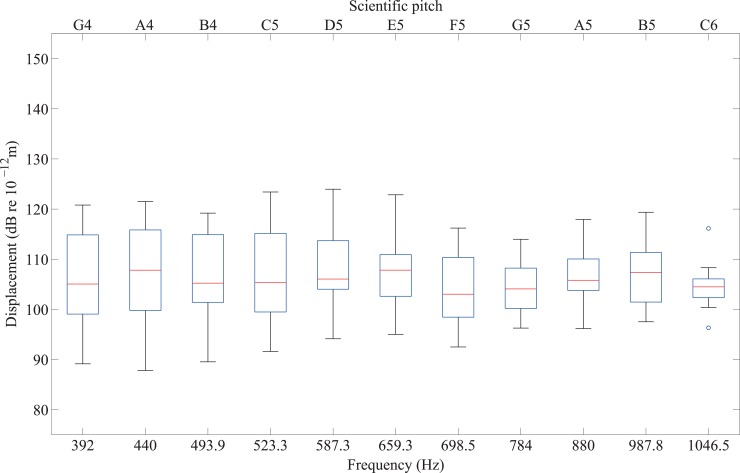
Vibrotactile thresholds on the fingertip from 14 participants with normal hearing for notes between G4 and C6.

The boxplots in Fig 6 represent the combined thresholds of 14 participants with normal hearing for the white notes only between G4 and C6. In this high-frequency range the median threshold is found to be comparatively flat, typically lying between 100 and 110dB.

**Fig 7:**
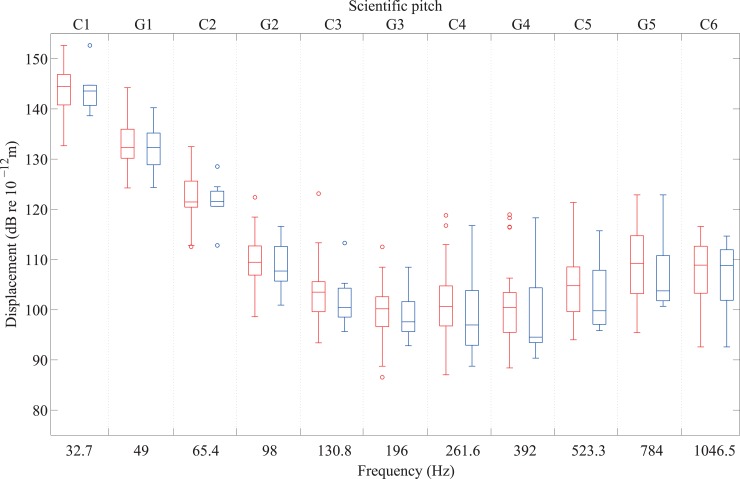
Vibrotactile thresholds on the fingertip from 32 participants with normal hearing (left-hand box in red) and 8 participants with hearing impairments (right-hand box in blue) for notes between C1 and C6.

In Fig 7, the thresholds of 8 participants with a severe or profound hearing loss can be compared with those of the 32 participants with normal hearing across the five octaves from C1 to C6. It is seen that the interquartile ranges of the two groups overlap and the results of the Mann-Whitney tests for each note in Table 1 showed no significant differences between the mean thresholds of the two groups for each note.

**Table 1:** Mann-Whitney test results to compare thresholds on the middle fingertip from participants with normal hearing and with a severe or profound hearing loss.

Note^a^	*U*^b^	*r*	*p*
C1	121.000	−0.040	0.813
G1	108.000	−0.110	0.499
C2	112.000	−0.090	0.588
G2	113.000	−0.080	0.612
C3	121.000	−0.040	0.813
G3	125.000	−0.020	0.919
C4	103.000	−0.130	0.398
G4	80.000	−0.260	0.105
C5	108.000	−0.110	0.499
G5	99.000	−0.160	0.327
C6	81.000	0.000	1.000

^a^ Observations, *N* = 40 except for C6 (*N* = 33).

^b^ Mann-Whitney test statistic.

### Experiment I (forefoot and heel)

**Fig 8:**
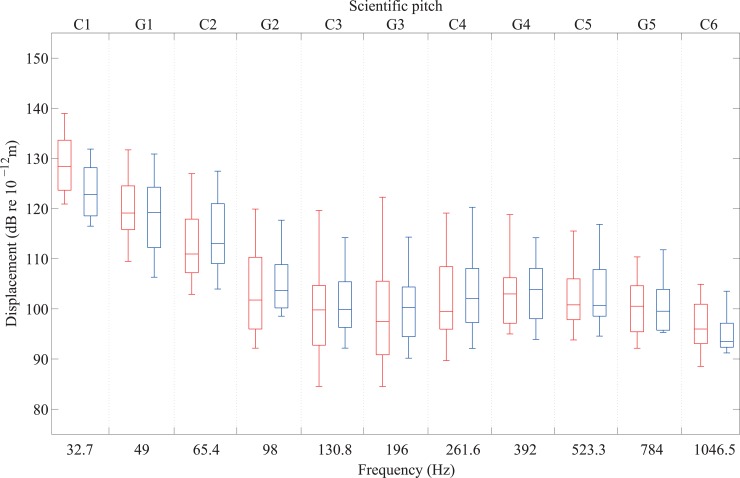
Vibrotactile thresholds on the forefoot (left-hand box in red) and heel (right-hand box in blue) from 20 participants with normal hearing for notes between C1 and C6.

The individual vibrotactile thresholds of 20 participants with normal hearing are shown in Fig 8 for 11 notes between C1 and C6. Of the 20 participants who were tested on the forefoot, one participant could not feel G5 and two participants could not feel C6. Of the 20 participants that were tested on the heel, one participant could not feel G5 and four participants could not feel C6. Table 2 shows that no significant differences were found between thresholds for forefeet and heels (independent *t*-test, *p*>0.05), except for C1 which showed a large effect (*r* = 0.44). The thresholds for the forefoot and heel do not show the distinct U-shape curve seen for the fingertip. Relatively few published studies report the use of large contactors to determine thresholds for the foot, and none at frequencies above 315Hz [37,38,39]. The closest comparator study is by Morioka and Griffin [39] where thresholds were measured using a rigid sloping footrest for the entire sole of the foot (i.e. forefoot and heel); hence a comparison can be made with these thresholds which correspond to displacement in the vertical direction.

**Table 2:** Independent *t*-test results to compare thresholds on the heel and forefoot.

Note	*t*(38)^a^	*r*	*p*
C1	3.053	0.444	0.004
G1	0.922	0.148	0.362
C2	−0.777	0.125	0.442
G2	−0.542	0.088	0.591
C3	−0.308	0.050	0.760
G3	−0.466	0.075	0.644
C4	−0.262	0.042	0.794
G4	−0.145	0.024	0.885
C5	−0.296	0.048	0.769
G5	−0.425	0.071	0.673
C6	0.887	0.155	0.382

^a^ Degrees of freedom, *df* = 36 for note G5 and *df* = 32 for C6.

**Fig 9:**
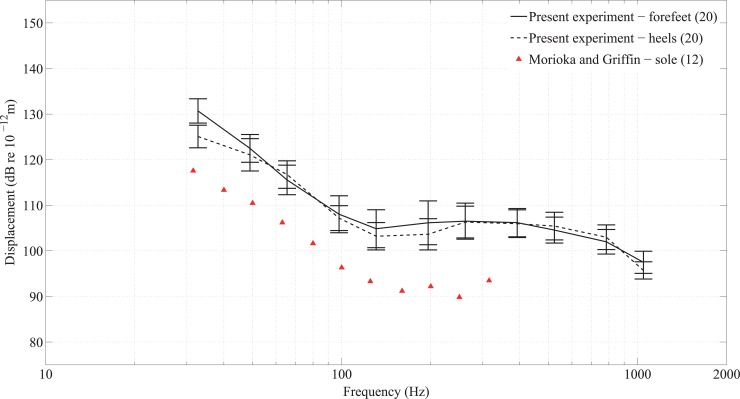
Comparison of vibrotactile thresholds on the forefoot and heel from the present experiment with other published data from participants with normal hearing. The number of participants is indicated in brackets. The results from the present experiment are shown as mean values with 95% confidence intervals, and the results from Morioka and Griffin are median values.

The mean thresholds of these participants are compared in Fig 9 with those measured on the sole of the foot by Morioka and Griffin [39].The shapes of the curves from Morioka and Griffin and the present experiment are similar, but the thresholds for the entire sole are significantly lower than for the forefoot or heel. This could be explained by spatial summation from the Pacinian receptors over the larger area of the sole of the foot but it could also be due to differences in the methodology used to determine the thresholds. Morioka and Griffin also used an up-down algorithm but unlike the present experiment they used a light to inform the participant when the stimulus was presented. This could partly explain the lower thresholds so it would be beneficial for future research to determine the differences between thresholds on the forefoot, heel and entire sole using the same psychophysical method.

**Fig 10:**
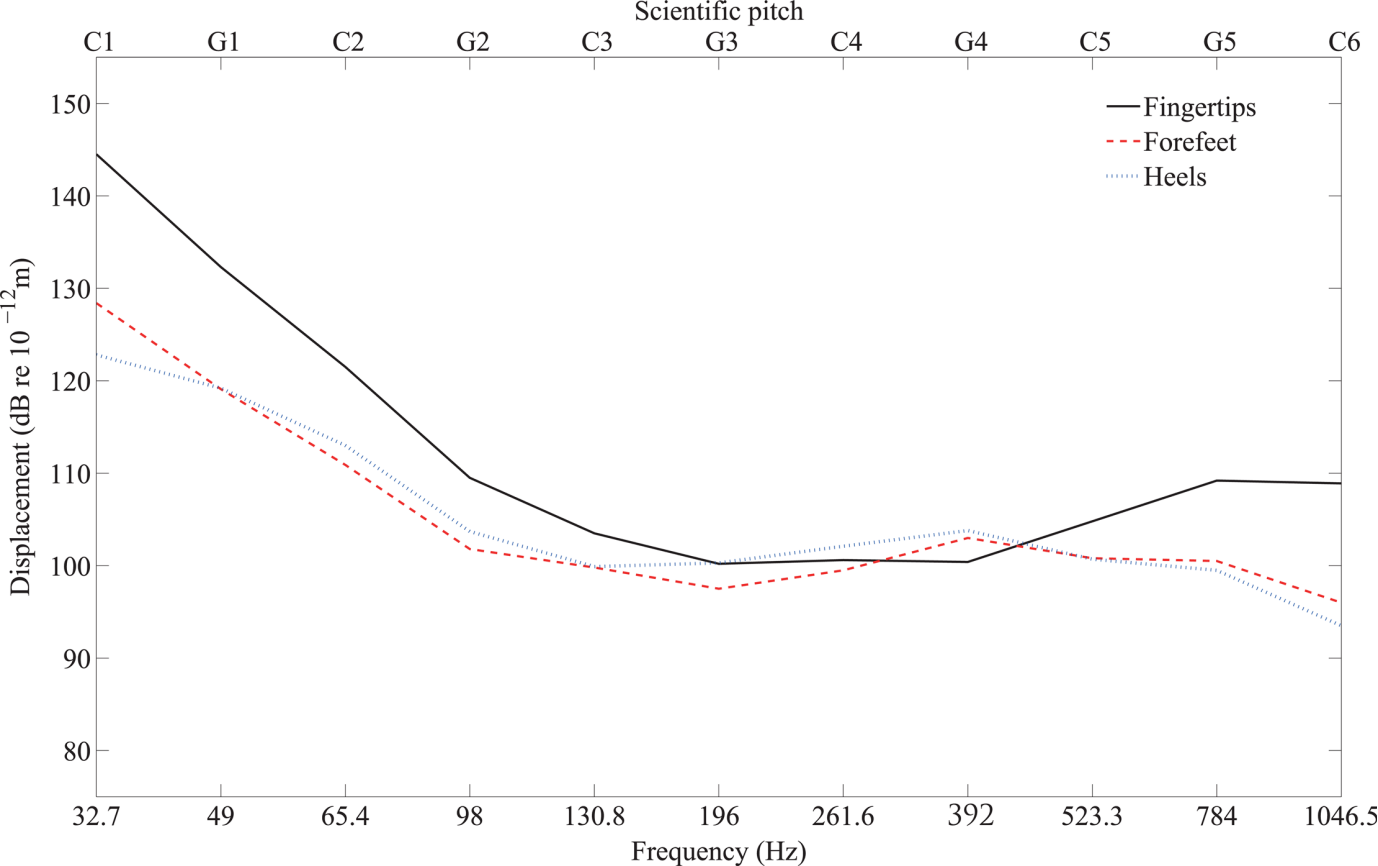
Comparison of the median threshold values from fingertip, forefoot and heel for notes between C1 and C6 from participants with normal hearing.

A comparison is shown in Fig 10 of the median thresholds on fingertip, forefoot and heel for the 11 notes across five octaves between C1 and C6 using the different sizes of contactors described in section 2.3.1 and 2.3.2. Table 3 shows that the thresholds were significantly lower for the forefoot than the fingertip between C1 and C3 inclusive and G5 and C6 (Mann-Whitney, *p*<0.05). However, there was no significant difference between thresholds of fingertips and forefeet (Mann-Whitney test, *p*>0.05) in the range G3 to C5 (inclusive). Table 4 shows that there was a significant difference between the mean thresholds for fingertips and heels (independent *t*-test, *p*<0.05), except between C3 and C5 inclusive.

**Table 3:** Mann-Whitney test results to compare thresholds on the middle fingertip and forefoot.

Note^a^	*U*	*r*	*p*
C1	9.000	−0.810	0.000
G1	35.000	−0.740	0.000
C2	90.000	−0.600	0.000
G2	179.000	−0.370	0.008
C3	213.000	−0.280	0.044
G3	293.000	−0.070	0.612
C4	298.000	−0.060	0.679
G4	236.000	−0.220	0.114
C5	248.000	−0.190	0.176
G5	94.000	−0.570	0.000
C6	45.000	−0.680	0.000

^a^ Observations, *N* = 52 except for note G5 (*N* = 51) and C6 (*N* = 45).

**Table 4:** Independent *t*-test results to compare thresholds on the middle fingertip and heel.

Note	*t*(50)^a^	*r*	*p*
C1	14.892	0.903	0.000
G1	8.664	0.775	0.000
C2	5.129	0.587	0.000
G2	3.178	0.410	0.003
C3	1.900	0.259	0.063
G3	−0.549	0.077	0.586
C4	−1.029	0.144	0.308
G4	−1.085	0.152	0.283
C5	0.979	0.137	0.332
G5	4.270	0.521	0.000
C6	7.265	0.750	0.000

^a^ Degrees of freedom, *df* = 49 for note G5 and *df* = 41 for C6.

It should be noted that the contact force applied by the fingertip, heel or forefoot on the contactor was not controlled in these experiments. ISO 13091–1 [18] requires use of a 0.4cm diameter contactor (±0.21cm) to be used when taking fingertip measurements to assess nerve dysfunction. With such a narrow contactor, an increased contact force can lead to lower thresholds at higher frequencies (e.g. [33]) for which there can be significant skin indentation. In the present study participants maintained a relaxed hand position using a much larger contactor so the results should not have been affected by variations in contact force.

**Fig 11:**
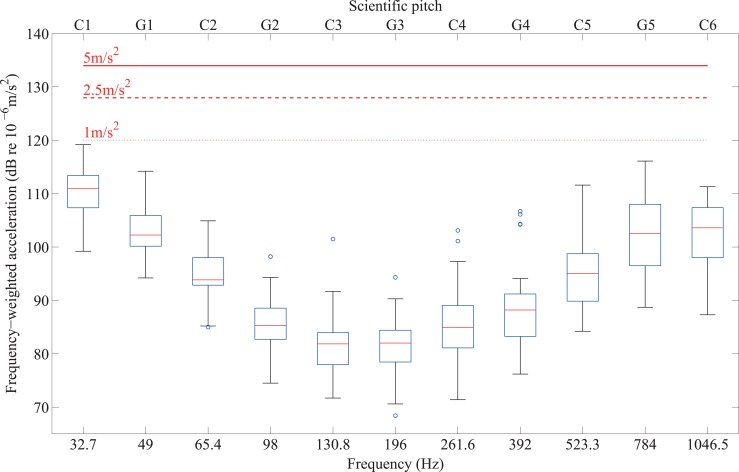
Vibrotactile thresholds on the fingertip from 32 participants with normal hearing for notes between C1 and C6 in terms of the frequency-weighted acceleration for comparison with values of 1m/s^2^ rms (equivalent to 120dB), 2.5m/s^2^ rms (equivalent to 128dB) and 5m/s^2^ rms (equivalent to 134dB).

**Fig 12:**
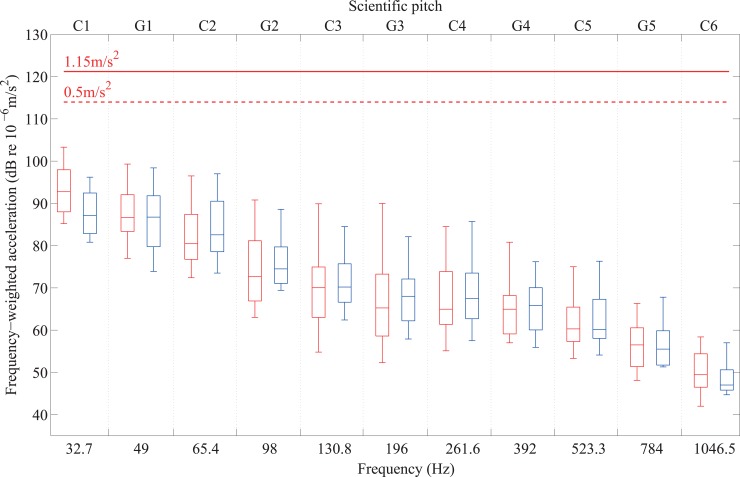
Vibrotactile thresholds on the forefoot (left-hand box in red) and heel (right-hand box in blue) from 20 participants with normal hearing for notes between C1 and C6 in terms of the frequency-weighted acceleration for comparison with values of 0.5m/s^2^ rms (equivalent to 114dB) and 1.15 m/s^2^ rms (equivalent to 121.2dB).

Figs 11 and 12 show the thresholds of the 32 participants with normal hearing for each of the 11 notes from C1 to C6 in terms of frequency-weighted acceleration. These are shown for fingertips on Fig 11 using the weighting for hand-arm vibration [40], and for forefeet and heels on Fig 12 using the weighting for whole-body vibration [41]. In Great Britain, health and safety at work regulations [42] define a daily exposure limit value which is the maximum amount of vibration to which someone may be exposed to on any single day, and a daily exposure action value above which action is required to reduce exposure. For hand-arm vibration, the daily exposure limit value is 5m/s^2^ rms A(8) and the daily exposure action value is 2.5m/s^2^ rms where A(8) indicates time-weighted exposure for a reference duration that corresponds to an 8 hour day. The ‘exposure limit value’ gives the level of daily exposure which must not be exceeded whereas the ‘exposure action value’ gives the level of daily exposure which, if reached or exceeded, requires action to be taken to reduce the risk. In addition, a limit value for hand-arm vibration of 1m/s^2^ rms is suggested for which vascular symptoms will not usually occur [43]; note that this is not a time-weighted value. For whole-body vibration, which applies to the forefoot and heel, the daily exposure limit value is 1.15m/s^2^ rms A(8) and the daily exposure action value is 0.5m/s^2^ rms A(8). An assessment of the dynamic range available when music is presented in the form of vibrotactile stimuli can be made on the basis that the presentation level in terms of frequency-weighted acceleration should not exceed the daily exposure action value (and additionally for fingertips, not exceed the limit value of 1m/s^2^ rms). The results show that the thresholds are between 17dB and 58dB below levels associated with any health risk; hence it is feasible to consider vibrotactile presentation of music well-above threshold.

### Experiment II (fingertip)

**Fig 13:**
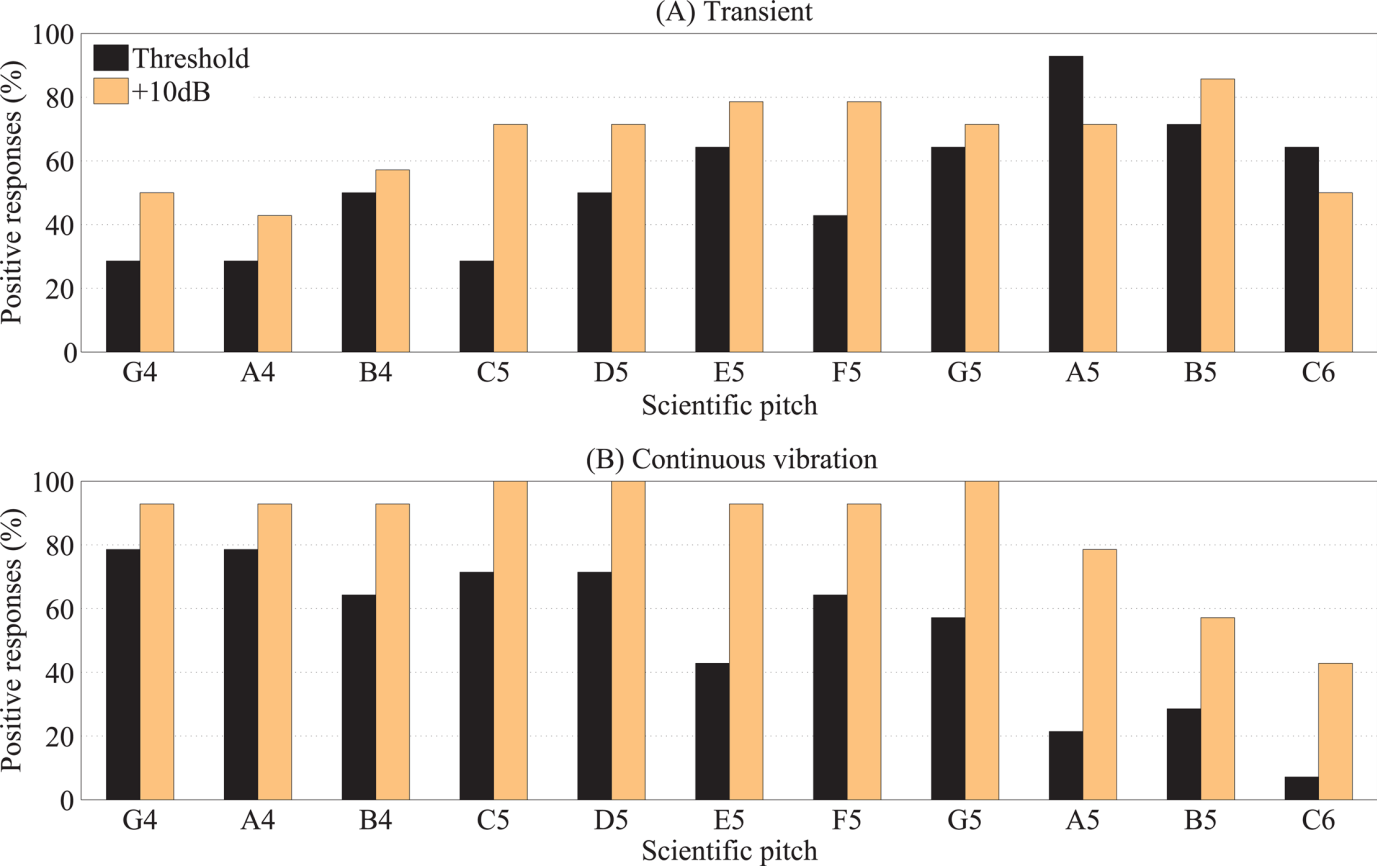
Percentage of 14 participants with normal hearing responding positively that (A) transient vibration at the beginning or end of the note could be felt and (B) continuous vibration of the note could be felt via the middle fingertip.

Fig 13 shows the responses for the perception of transient and continuous high-frequency vibration. Participants’ awareness of transient vibration at the beginning and/or end of any of the notes increased with increasing pitch height, peaking at A5 (880Hz) and B5 (987.8Hz). Conversely, and therefore as expected, participants’ awareness of the continuous parts of the notes was relatively high for the lower pitches in the range, decreasing at A5 and B5 where transient awareness peaked. Participants were typically more aware of the transient parts of each note when presented 10dB above threshold compared to at threshold. For notes between G4 (392Hz) and G5 (784Hz), 93.7% of participants, on average, responded positively that they could feel continuous vibration when presented with the stimuli at 10dB above threshold. However, when the notes were presented at threshold level, four of the 14 participants reported not being able to feel C6 (1046.5Hz) and one participant reported not being able to feel B5 (987.8Hz).

## Discussion

### Vibrotactile thresholds

The duration of the signals used in the present study was 1s, representing a minim (half note) at 120bpm. For the practical purposes of presenting music in the form of vibrotactile stimuli it is necessary to consider the extent to which the thresholds reported above would apply to the same notes played for longer or shorter durations. Based on Zwislocki’s theory and its experimental validation by Verrillo [25] for sinusoids at and below 500Hz, thresholds would not be expected to differ for longer durations, but the threshold would be expected to be raised towards the theoretical maximum value for shorter durations. To put this in context for music at 120bpm, a quaver (eighth note) and a semiquaver (sixteenth note) have durations of 0.25s and 0.125s respectively so the thresholds would be raised by a maximum of ≈2dB and ≈4dB respectively. These are small rises in the context of inter-participant variation. It is reasonable, therefore, to assume that the thresholds found in the present study would apply between C1 and C5 (i.e. up to ≈500Hz) for notes no shorter than a semiquaver at tempos up to a maximum of 120bpm.

Thresholds for notes between C1 and G2, and notes G5 and C6, presented to the forefoot and heel were significantly lower than the fingertip. This could be attributable to spatial summation by Pacinian receptors because the contactors used for the forefoot and heel were larger than those used for the fingertip [16]. However, it may also be attributable to the receptive fields for Pacinian receptors on the foot which are more evident on the forefoot than the heel [44]. As vibrotactile presentation to forefeet and heels is a practical option for many instrumental musicians, the lower thresholds at low and high-frequencies are useful as they increase the available dynamic range. It was expected that some participants would not be able to feel some of the high notes because thresholds were determined at frequencies higher than are normally considered for medical purposes. However it was only G5 and C6 that could not be felt by a few participants; hence the vibrotactile presentation of notes above C6 is unlikely to be a feasible solution.

Higher percentages of tests on the heel (100%) and forefoot (87%) were valid than tests on the fingertip (78%). It was only for the heel that the repeat test of C4 was always within ±4dBV of the first measurement and the skin temperature was always within the acceptable range during the entire experiment. For the purposes of obtaining reliable measurements the implication is that the heel is most reliable and the fingertip is least reliable. Future research could investigate whether there are differences between pitch perception at these three locations on the body.

No significant differences were found between the thresholds of participants with normal hearing and with a severe or profound hearing loss when notes between C1 and C6 were presented to the fingertip. While this finding cannot be interpreted as indicating that the thresholds of participants with and without hearing impairments are the same, they nevertheless confirm the results of two previous studies using the glabrous skin of the fingers. However, these tested vibrotactile thresholds for signals only up to 300Hz, as opposed to 1046.5Hz in the present study. Bernstein *et al* [45] compared the thresholds between 10Hz and 250Hz on the index finger of two children aged 9 and 10 years who were profoundly deaf with those of other children and found no significant difference. Similarly, Moallem *et al* [46] compared thresholds between 2Hz and 300Hz on the glabrous skin of the thumb and index finger of adults with normal hearing and those who were congenitally deaf, and found no difference. The results of the present study also support those found in two previous studies using other parts of the body. Using the hairy skin of the inner side of the forearm, Lamoré [47] found similar thresholds at 500Hz and 1kHz (approximately C5 and C6 respectively) for adults with normal hearing and a severe hearing loss. With the inner and outer wrist, Donahue and Letowski [48] found no significant difference between five adults with normal hearing and five adults with a severe or profound hearing loss when comparing vibrotactile thresholds between 32Hz and 500Hz.

### Perception of transient and continuous high-frequency vibration

In Experiment II participants with normal hearing were asked to report their perception of transient and continuous high-frequency vibration for notes from G4 to C6 inclusive that were presented at, and 10dB above each participant’s individual vibrotactile threshold for each note. More participants reported being able to feel the continuous signal when it was presented to the fingertip at a level of 10dB above threshold, than at their individual threshold. The continuous signal represents pitch, so this finding is important as it suggests that musicians are more likely to be able to detect pitch if the signal is presented at least 10dB above threshold. In addition, more participants reported being aware of the transient parts of each note when presented to the fingertip at a level of 10dB above threshold than when presented at threshold. The onset and decay behaviour of a note played on a musical instrument is usually more complex than that of a sinusoidal signal, and can provide information as to the instrument being played, so this finding is also important. Whilst vibrotactile feedback might not be sufficiently informative for musicians to detect subtle differences, they are more likely to be able to perceive that different instruments are being played if the signal is presented at least 10dB above threshold.

### Implications for the vibrotactile presentation of music

For the purposes of determining the potential for the vibrotactile presentation of music the range of musical notes that can be perceived and their dynamic range can now be assessed on the basis of the results from Experiments I and II. The available dynamic range is defined here as the lowest level of vibration at which it is practical to feel and use vibrotactile feedback up to the highest vibration level to which the human body can be exposed with minimal or no risk. An assessment of the available dynamic range is based on the assumption that the vibrotactile presentation level in terms of frequency-weighted acceleration should not exceed the daily exposure action value, and additionally for fingertips, not exceed the limit value of 1m/s^2^ rms. Thresholds for the fingertip, forefoot and heel were found to be sufficiently low for presentation at threshold itself not to carry any health risk, but, realistically, in the context of practice, rehearsal and performance, the presentation level would almost always be above threshold so that musicians would not have to concentrate on sensations close to, or at threshold level, whilst singing or playing. The main implication of the results of Experiment II is that musicians are more likely to be able to detect pitch and identify different instruments if signals are presented at least 10dB above threshold. Hence when assessing the available dynamic range based on vibrotactile thresholds for the fingertip from Experiment I, it is appropriate to consider presentation levels that are 10dB above the median threshold values in Figs 11 and 12 and compare these with the limit values that would avoid any health risk. However, to define the range of musical notes that could be presented in the form of vibrotactile stimuli, it is necessary to consider the reduction in the awareness of the continuous parts of high notes above G5 found in Experiment II. This has implications for pitch perception because detecting only the onset of a musical note does not provide the information needed to identify its pitch. In addition, some participants could not feel C6 at all, irrespective of whether it was presented to the fingertip, forefoot or heel. For the purpose of vibrotactile presentation of pitch, C1 to G5 is likely to be the most realistic range. For signals presented 10dB above the median threshold between C1 and G5, the available dynamic range would be at least 7dB and at most 36dB for the fingertip, at least 11dB and at most 47dB for the forefoot, and at least 17dB and at most 48dB for the heel.

Whilst this dynamic range should be ample for many types of instrument and musical genre, there are reasons to consider limiting the range using compression. For example, Békésy [49] highlighted potential problems with vibrotactile pitch perception when he presented sinusoidal signals of 25Hz, 50Hz and 100Hz that were 20dB above threshold, and found that an increase in amplitude corresponded to the subjective assessment that the pitch had decreased. Békésy [50] subsequently carried out further experiments with the intention of trying to understand more about the relationship between auditory and vibrotactile perception. These showed that pitch perception from vibration on the skin depended on both the frequency and magnitude of the stimulus. He found that the sensation of pitch was not purely determined by neural discharges synchronous with the frequency of the stimulus, but also on other neural interactions. Békésy’s experiments led him to consider the sensation of pitch via the skin not only as a function of the excitation frequency but of several other factors including the magnitude and area of the excitation, as well as adaptation to the stimuli. Morley and Rowe [51] noted that Békésy’s observations were made in 1957 before precise data on the response characteristics of mechanoreceptor classes were available, and are difficult to comprehend in the light of more recent neurophysiological information. In fact, Morley and Rowe found different effects of amplitude on pitch perception in their experiments. They presented 30Hz or 150Hz sinusoids that were 10dB above threshold followed by the same frequency at levels up to 50dB above threshold and found so much inter- and intra-subject variability in the effect of amplitude on perceived pitch that they could not draw definitive conclusions. Further reasons to avoid levels that are over 40dB above threshold are that the perceived intensity on the fingertip decreases with increased time of stimulation [52] and exposure to levels between 20dB and 50dB above threshold at frequencies of 2Hz, 20Hz and 200Hz temporarily increases the threshold due to a depression in the excitability of the mechanoreceptors [53]. There would be no benefit in using such high levels of vibrotactile presentation if they reduced a musician’s ability to perceive musical dynamics. Future research could address the issue of pitch perception in terms of relative pitch based on the proposed levels for vibrotactile presentation reported in this paper.

## Conclusions

This study determined vibrotactile thresholds in order to assess the potential for vibrotactile presentation of music to the glabrous skin of the fingertip, forefoot and heel. Based on a 1s signal corresponding to a minim (half note) at 120bpm, the main findings are as follows:

1. Vibrotactile thresholds for the fingertip from participants with normal hearing and a severe or profound hearing loss show the characteristic U-shape curve for notes between C1 and C6. More detailed measurements using participants with normal hearing for notes between G4 and C6 indicate that the curve is relatively flat at these high frequencies. Vibrotactile thresholds for the forefoot and heel did not show the characteristic U-shape curve. Compared to the fingertip, the forefoot had lower thresholds between C1 and C3, and the heel had lower thresholds between C1 and G2; this is attributed to spatial summation by the Pacinian receptors over the larger contactor area used for the forefoot and heel.
2. No statistically significant differences were found between the vibrotactile thresholds for notes between C1 and C6 presented to the fingertip of participants with normal hearing and with a severe or profound hearing loss.
3. Participants with normal hearing were typically more aware of the transient vibration at the beginning and/or end of any of the notes between G4 and C6 when stimuli were presented 10dB above threshold, rather than at threshold. For notes between G4 and G5, an average of 94% of participants responded positively that they could feel continuous vibration when presented with the stimuli at 10dB above threshold.
4. The range of musical notes that can be considered for the vibrotactile presentation of music is from C1 to G5. The available dynamic range has been estimated based on the avoidance of health problems associated with exposure to vibration. Assuming vibrotactile presentation is at least 10dB above threshold the available dynamic range is at least 7dB and at most 36dB for the fingertip, at least 11dB and at most 47dB for the forefoot, and at least 17dB and at most 48dB for the heel. Although the range of notes is more limited than with human hearing, the range of fundamental frequencies for the human voice and many instruments lie within this range. The dynamic range thus available for the vibrotactile presentation of music would be sufficient for most instruments and musical genres, although compression may be needed to limit the negative effect of amplitude on vibrotactile pitch perception.
